# Epidemiology of Infections Caused by Four Emerging *Vibrio* Species of Concern Reported to the Cholera and Other Vibrio Illness Surveillance System (COVIS), United States, 1988–2024

**DOI:** 10.1016/j.jfp.2026.100891

**Published:** 2026-08-11

**Authors:** Daniel L. Weller, Marisa Hast, Robin P. Pendley Louis, Christine C. Lee, Michael J. Hughes

**Affiliations:** Division of Foodborne, Waterborne, and Environmental Diseases, National Center for Emerging and Zoonotic Infectious Diseases, U.S. Centers for Disease Control and Prevention, Atlanta, Georgia

**Keywords:** V. alginolyticus, V. cholerae, V. fluvialis, V. mimicus, Vibriosis, Waterborne disease

## Abstract

**Background:**

Incidence of vibriosis infections caused by several emerging *Vibrio* species has increased in the United States, including *V. alginolyticus* (Va), *V. cholerae* (Vc), *V. fluvialis* (Vf), and *V. mimicus* (Vm), which contribute substantially to overall vibriosis burden. The objective of this analysis was to characterize the epidemiology of these emerging *Vibrio* species using US national surveillance data.

**Methods:**

We conducted an analysis of national Cholera and Other Vibrio Illness Surveillance (COVIS) data from 1988 to 2024. Spline regression was used to estimate trends. Counterfactual random forests were used to compare epidemiologic and clinical profiles across species.

**Results:**

During 1988–2024, 4,350 Va, 2,856 Vc, 1,722 Vf, and 582 Vm infections were reported, and incidence increased for all species and varied by region. The largest relative increases were for Vf (+101%), Vc (+79%), and Va (+67%). Va had an epidemiologic profile distinct from Vc, Vf, and Vm; Va infections were associated with persons aged 1–17 years, waterborne transmission, and wound and ear infections. Vc, Vf, and Vm infections were more often foodborne and associated with gastrointestinal illness. Hospitalization was more common for Vc, Vf, and Vm (37–42%) compared with Va infections (13%).

**Conclusions:**

Va, Vc, Vf, and Vm infections represent a growing component of vibriosis infections in the United States and exhibited distinct epidemiologic profiles with substantial morbidity. Vibriosis prevention efforts should be tailored to include both foodborne and non-foodborne transmission in regions and populations at higher risk, and clinicians should have increased awareness of emerging *Vibrio* species.

**Plain Language:**

*Vibrio* are naturally occurring bacteria that can cause illnesses ranging from gastroenteritis to serious wound infections. Infections caused by four emerging *Vibrio* species of concern, namely *V. alginolyticus* (Va), *V. cholerae* (Vc), *V. fluvialis* (Vf), and *V. mimicus* (Vm), have been increasing in the United States. During 1988–2024, the incidence of infections caused by these species increased. Va infections were distinct from the Vc, Vf, and Vm infections. Va infections were more often associated with waterborne transmission, people aged 1–17 years, and ear and wound infections. Vc, Vf, and Vm infections were more often foodborne and linked to gastroenteritis and hospitalization. These species represent a growing component of vibriosis in the United States and have distinct clinical and epidemiologic profiles. Prevention efforts should be tailored to address foodborne and non-foodborne transmission in populations at higher risk for specific infection types.

*Vibrio* is a genus of free-living aquatic bacteria that dwell primarily in salty or brackish water and can infect humans through consumption of contaminated food or through skin contact with contaminated water or marine life (Gherlan et al., 2025; Igbinosa & Okoh, 2010; Jacobs Slifka et al., 2017; Muller et al., 2016; Mustapha et al., 2013; Ramamurthy et al., 2014). Vibriosis research and prevention in the United States have focused on *V. parahaemolyticus* (Vp) and *V. vulnificus* (Vv), which account for most reported illnesses and mortality (Hast et al., 2026; Newton et al., 2012). However, other *Vibrio* species, including *V. alginolyticus* (Va), *V. cholerae* (Vc), *V. fluvialis* (Vf), and *V. mimicus* (Vm), contribute to the overall vibriosis burden in the United States and cause a wide spectrum of illness, including gastroenteritis, skin and soft tissue infection (SSTI), and bacteremia (Shah et al., 2024; Pérez-Duque et al., 2021). Va incidence is greater than Vv, while the incidence of infections caused by Vc and Vf is increasing faster than any other *Vibrio* species in the United States (Shah et al., 2024; Christopher et al., 2006). This highlights the need for improved understanding of their epidemiology, and for laboratory surveillance for accurate speciation, which is essential for informing proactive prevention strategies.

Available studies and outbreak reports suggest that these *Vibrio* species differ in their ecology, virulence factors, clinical characteristics, and routes of transmission (León Robles et al., 2013; Pérez-Duque et al., 2021; Sampaio et al., 2022). Prevention and surveillance strategies that focus on Vp and Vv may fail to address the full spectrum of *Vibrio* hazards. While there are data available to guide Va, Vc, Vf, and Vm infection prevention in the United States, these analyses are few and often reflect surveillance data from over a decade ago, individual case studies, or are limited to specific regions or states (Alam et al., 2023; Jacobs Slifka et al., 2017; Morgado et al., 2024; Newton et al., 2012; Sodders et al., 2025). The most recent study describing Va infection in the United States analyzed Cholera and Other Vibrio Illness Surveillance (COVIS) data from 1988 to 2012 (Jacobs Slifka et al., 2017). Since then, changes in the vibriosis case definition, the adoption of culture-independent diagnostic tests (CIDTs), and whole genome sequencing (WGS) have changed enteric illness diagnosis and surveillance, including pathogen identification, speciation, and sub-typing (Foodborne Disease Burden Epidemiology Reference Group, 2023; Kwong et al., 2015; Ray et al., 2024; Council of State and Territorial Epidemiologists, 2017; Council of State and Territorial Epidemiologists, 2012). These shifts may affect the detection and reporting of specific *Vibrio* species.

These gaps highlight the need for an updated, comprehensive assessment of the epidemiology of Va, Vc, Vf, and Vm infections in the United States, in comparison to Vp and Vv. COVIS data are well suited to address this need, given their national scope and detailed case data spanning more than three decades. We used COVIS data from 1988 to 2024 to provide an updated assessment of Va, Vc, Vf, and Vm epidemiology in the context of evolving environmental and diagnostic landscapes.

## Methods

### Data.

COVIS data from 1988 to 2024 were analyzed. For all culture-confirmed infections caused by bacteria in the *Vibrionaceae* family, state and local health officials interview patients using a nationally standardized case report form. This captures information on diagnostic laboratory results, clinical outcomes, underlying health conditions, and exposures to seafood and bodies of water in the seven days before illness onset. Officials then transmit these data to CDC. Once data are received, CDC identifies the presumptive transmission route for each case (e.g., foodborne, not foodborne).

Data for all Va, Vc (toxigenic and non-toxigenic), Vf, and Vm, as emerging *Vibrio* species of concern, and Vp and Vv, as comparison species, were used here.

### Descriptive analyses.

All analyses were implemented in R (version 4.4.1). Vibriosis has been reported to the CDC since 1988 but did not become a nationally notifiable condition until 2007 (Council of State and Territorial Epidemiologists, 2007), so incidence analyses were limited to 2007–2024 due to difficulty ascertaining appropriate denominator data prior to 2007. Denominator data were obtained from the U.S. Census Bureau. The number and proportion of ill-persons with specific exposures, symptoms, clinical outcomes, and related characteristics were summarized for each species across the entire analysis period (1988–2024). Average annual state-level incidence during 2007–2024 was visualized using the usmap, sf, and ggplot2 packages (Di Lorenzo, 2025; Hadley Wickham, 216 C.E.; Pebesma, 2018; Pebesma & Bivand, 2023; Wickham et al., 2016; Wickham, 2009).

Race data were analyzed in two ways. For descriptive analyses, race was treated as a categorical variable with mutually exclusive levels, including a separate multiracial level. For the counterfactual random forest (CFRF) analysis described below, race was treated as separate binary indicator variables (e.g., if the patient identified as Asian, if the patient identified as White), allowing ill-persons to identify with multiple categories. This approach was used to align descriptive analyses with census-based population denominators in the tables.

To identify cases with ear infections and ear-related symptoms, natural language processing of a free-text symptoms field was performed. The ear infection and ear-related symptom data represent presence only and can have values of ‘yes’ or ‘not reported’.

### Regional comparisons.

To compare regional differences in vibriosis incidence for each species and for *Vibrio* overall, separate Bayesian generalized additive mixed models with a Bernoulli likelihood and logit link were used. The outcome of interest was whether a state reported any vibriosis cases associated with each species in a given year. The linear predictor included two smooth terms:

1. a thin plate spline for year with five basis functions to capture long-term trends;
2. a thin plate spline for log-transformed state population with five basis functions to account for systematic differences related to state size.

Coastal status was parameterized using an indicator for whether a state was coastal or noncoastal, with an additional term that captured differences between the Atlantic (Connecticut, District of Columbia, Delaware, Georgia, Massachusetts, Maryland, Maine, North Carolina, New Hampshire, New Jersey, New York, Rhode Island, South Carolina, and Virginia), Gulf (Alabama, Florida, Louisiana, Mississippi, and Texas) and Pacific (Alaska, California, Hawaii, Oregon, and Washington) coast states. A random intercept for state was also included to allow baseline outcome levels to vary across states and to account for within-state clustering. Weakly informative, data-driven priors were used (Table S1). The model was fit with the brms package using four chains of 3,001 iterations (Bürkner, 2017, 2018). From the fitted model, we derived posterior distributions to summarize contrasts between coastal and noncoastal states, and among the three coastal regions.

### Trends analysis.

To model trends in incidence of confirmed Va, Vc, Vf, Vm, Vp, and Vv infections reported to COVIS during 2007–2024, Bayesian regression was implemented using the brms package (Bürkner, 2017, 2018). A single zero-inflated negative binomial hierarchical model was fit using four chains of 3,001 iterations each to estimate trends across species. To allow species and states with sparse data to borrow strength from those with more observations, the model included:

1. a global smooth of year using a penalized thin plate regression spline with 10 knots to capture trends shared across all species.
2. species-specific and state-specific smooth deviations were included using factor-smooth interactions with species (five knots) and state (three knots), respectively.

Species was included as a fixed effect, and state was included as a random intercept. A normal(−15, 0.5) prior was used on the intercept, a normal(0, 0.3) prior on the fixed effects, a normal(0, 0.1) on the random effect, and a normal(−1, 1) prior on the zero-inflation parameter. Exponential priors were used on the global [exponential(12)], state-specific [exponential(6)] and species-specific [exponential(8)] smooth terms. A gamma(20, 10) prior was used to control overdispersion.

Past studies have suggested that fewer enteric illnesses were reported during the COVID-19 pandemic (Armistead et al., 2022; Barkley et al., 2025; Collins et al., 2022; Dougherty et al., 2023; Liu et al., 2022; Ray et al., 2021). As a sensitivity analysis, the trends model was fit excluding and including data from 2020 and 2021.

To evaluate changes associated with the transition from culture-based diagnostics to CIDTs, we compared median annual incidence before (2007–2014) and after (2015–2024) syndromic gastrointestinal panels with a *Vibrio* target became commercially available.

### Counterfactual random forest (CFRF).

We used CFRFs to compare the epidemiology of infections caused by the four emerging species of concern with each other and with Vp and Vv by modifying a previously described approach (Ray et al., 2024; Weller et al., 2024). CFRF produces odds ratios (ORs) for individual variables while adjusting for all other variables in the model (Jackson et al., 2020). We fita CFRF to quantify the odds that an ill-person was infected with Va compared to Vc, Vf, and Vm (*35, 36, 57*). Va was selected as the reference because it appeared most different from Vc, Vf, and Vm in descriptive analyses. The features included in the model were time period, characteristics of patient state of residence (e.g., if it was on the Atlantic, Gulf, Pacific or non-coastal), demographics (age, race/ethnicity, sex), exposures (e.g., consuming seafood), transmission route, medical history (e.g., if the patient had any comorbidities), clinical symptoms and outcomes, season, and the type of sample(s) tested (e.g., stool, blood). Separate CFRFs were fit to quantify the odds that an ill-person was infected with either Vp or Vv compared to the four emerging species of concern as a sensitivity analysis.

## Results

**Figure 1.**
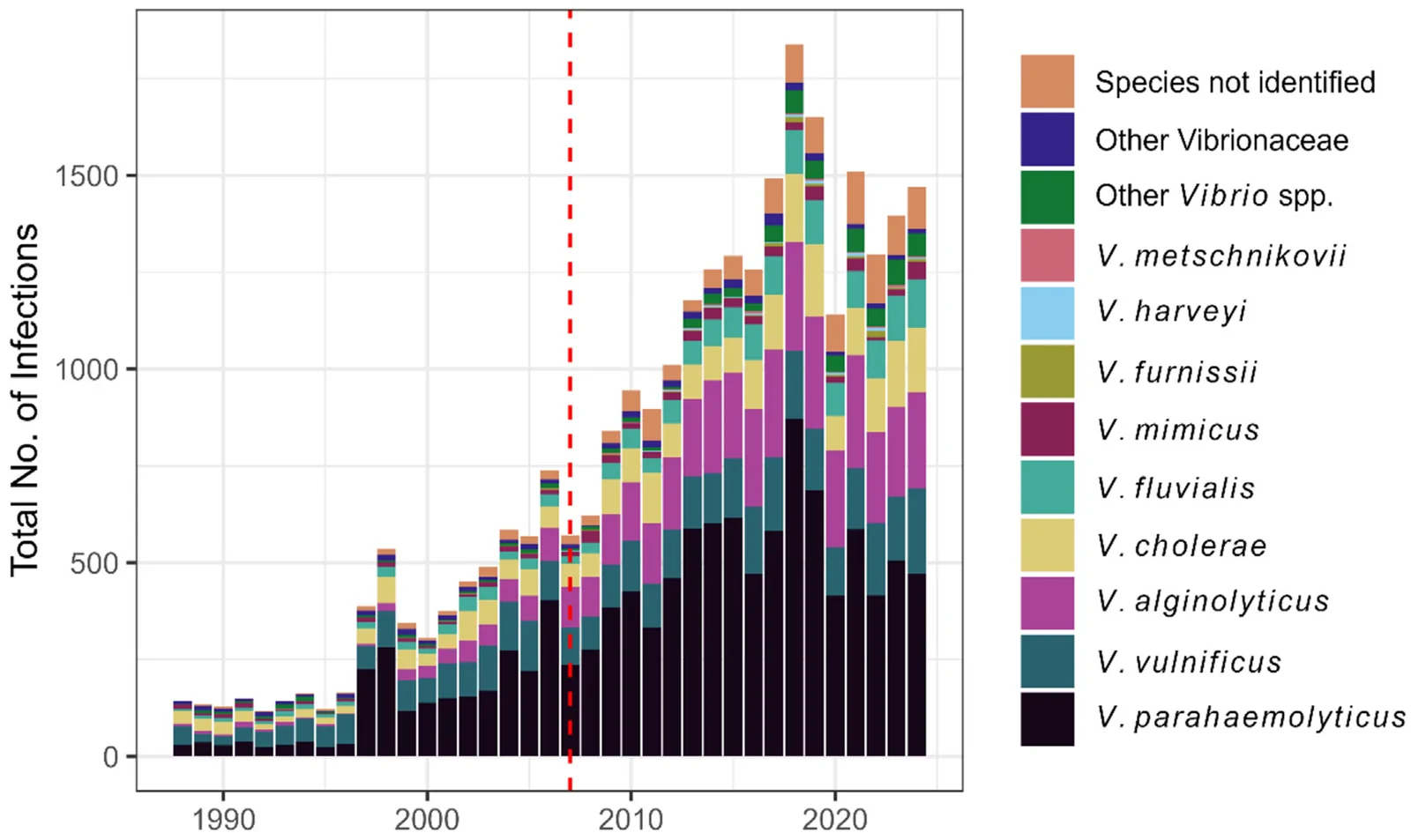
Number of culture-confirmed infections attributed to each *Vibrio* species reported to the Cholera and Other Vibrio Illness Surveillance System (COVIS) during 1988–2024. The red dashed line marks 2007, when vibriosis became nationally notifiable, and the COVIS catchment became the entire United States. “Species not identified” represents unspeciated *Vibrio* infections, while Other Vibrionaceae represents infections caused by other genera, namely *Grimontia hollisae* and *Photobacterium damselae*.

A total of 4,350 Va, 2,856 Vc, 1,722 Vf, and 582 Vm infections were reported to COVIS from 1988 to 2024, as well as 11,338 Vp and 3,990 Vv infections. Of the Vc infections, 2,557 were caused by non-toxigenic and 299 by toxigenic Vc; 85% (253/299) toxigenic infections were identified as cholera and 15% (46/299) as vibriosis. The number of illnesses caused by each species increased over the analysis period (Fig. 1).

### Incidence Rates per 100,000 persons (IRs).

Incidence of Va, Vc, Vf, and Vm varied by region and coastal vs. noncoastal state of patient residence (Table 1). Incidence for Va, Vc, Vf, and Vm infections was lowest in winter and highest in summer (Table 1). Vc, Vf, and Vm infection incidence was highest among persons aged ≥60 years, and Va infection incidence was highest among persons aged 5–17 years (IR = 0.107), followed by those aged ≥60 years (IR = 0.066). Incidence was higher among males for all species.

Incidence varied by race and ethnicity, and *Vibrio* species. Incidence was highest among people identifying as Native Hawaiian and Other Pacific Islander (NHPI) for all species. The second highest incidence was among people identifying as White for all species except Vm, for which incidence was second highest among Black and African American (BAA) persons. Vf and Vm incidence was approximately two times higher among non-Hispanic persons compared with Hispanic persons; there was no difference for Va and Vc.

Difference in incidence between persons of different races was most pronounced for Va; incidence of Va for NHPI people was 0.545/100,000 compared to 0.057 in the next highest group, White persons. Va infection was 2.6 times higher among White persons compared to BAA persons.

### Symptoms, comorbidities, and clinical outcomes.

During 1988–2024, reported hospitalization rates were 13% for Va, 42% for Vc, 37% for Vf, and 38% for Vm (Table 2). Vv hospitalization (84%) and mortality (25%) rates were highest. Death was reported for 1% of persons with Va, 4% with Vc, 2% with Vf, and 2% with Vm infection.

Symptom profiles differed across species (Table 2). Fever, muscle pain, and gastrointestinal symptoms (e.g., cramping, diarrhea, vomiting) were more frequent among persons infected with Vc, Vf, and Vm. Skin and soft tissue manifestations (e.g., bullae and cellulitis) were more frequent among persons infected with Va. Ear issues were almost exclusively reported by persons infected with Va (23% of Va infections compared to 1% of Vc, Vf, Vm, Vp, and Vv infections). The proportion of ill people reporting comorbidities varied (Table S2).

### Treatment and diagnostics.

Most infections (>80%) across species were diagnosed by culture (Table 2). Persons with Va infections most frequently had a wound or ‘other’ sample type collected, while persons with Vc, Vf, Vm and Vp infections most frequently had a stool sample. Antibiotics were prescribed for most cases regardless of species. The highest rate of antibiotic treatment was for Vv (83%) and Va (75%) infections.

### Exposures.

Foodborne transmission was most common for persons with Vp (79%), Vf (72%), Vm (71%), and Vc (68%) infections and was uncommonly reported by persons infected with Va (3%) and Vv (19%) (Table 3). Consumption of any seafood was four to seven times higher among persons infected with Vp (73%), Vm (60%), Vc (55%), Vf (53%), and Vv (44%) compared to Va (10%). Similar differences were observed for consumption of raw seafood and specific seafood commodities, such as oysters.

Non-foodborne exposures also differed across species. Contact with bodies of water was higher for Va (62%) and Vv (60%) compared to all other species (<30%). Wound exposure to water was more frequently reported among persons with Vv (47%) and Va (37%) infection compared to other species. Contact with marine life was higher for Vv (17%) compared to all other species (<9%).

### Regional Differences.

**Figure 2.**
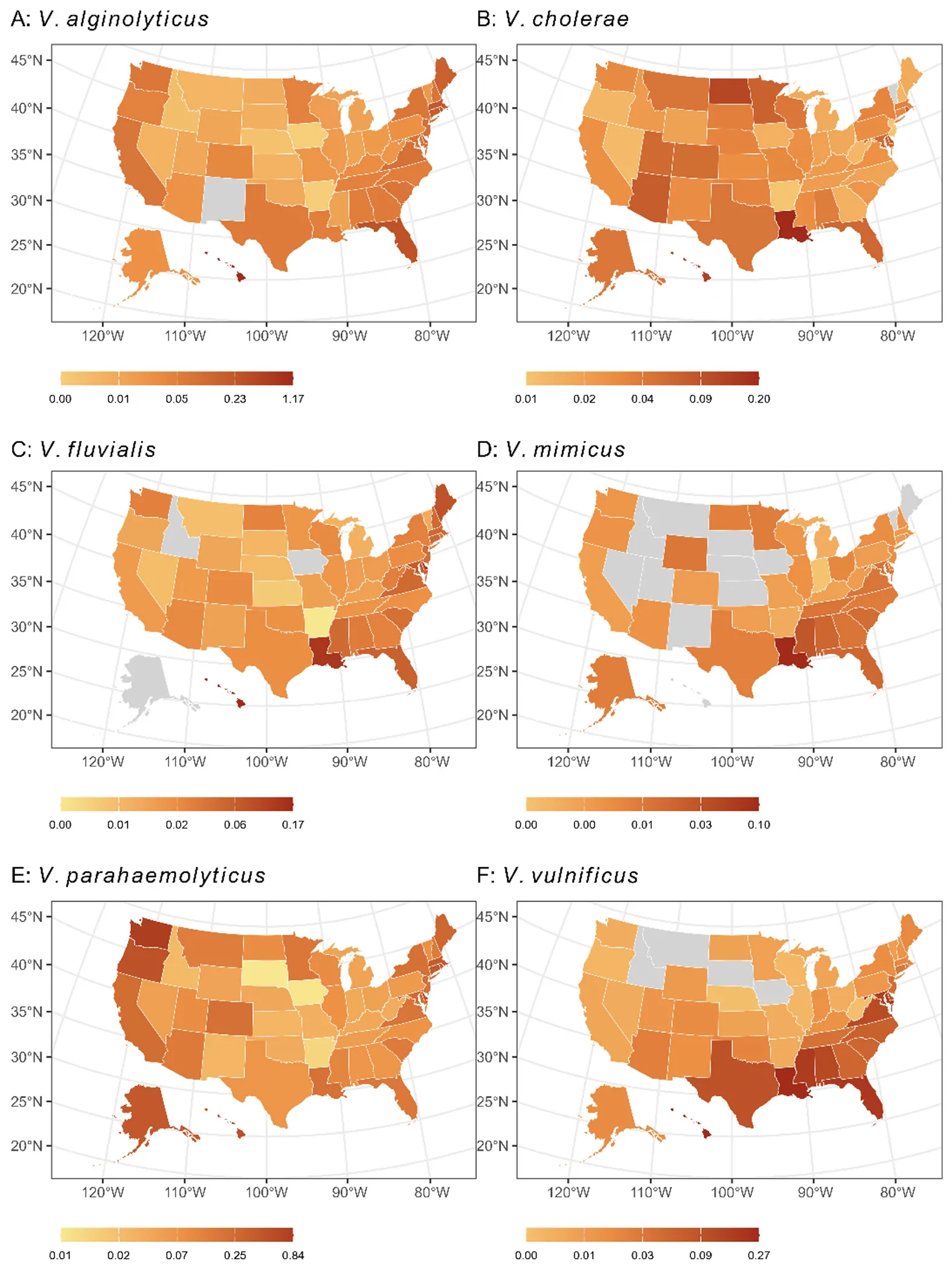
Average annual incidence of *Vibrio* infections per 100,000 people reported to the Cholera and Other Vibrio Illness Surveillance System (COVIS) during 2007–2024. Gray indicates no cases were reported in the given states. While vibriosis case data were transmitted to CDC during 1988–2006, vibriosis did not become a nationally notifiable condition until 2007. From 1988 through 2007, the number of reporting states increased steadily; however, because the COVIS catchment area was expanding during this period, it is not feasible to calculate incidence rates prior to 2007 due to the absence of a clearly defined denominator.

Certain states reported higher incidence for specific species. Hawaii reported a higher incidence of Va and Vf infection, while Louisiana reported a higher incidence of Vc, Vf, and Vm (Fig. 2). During 1988–2024, the probability a state reported ≥1 *Vibrio* infection in a given year varied by the state’s coastal status (Table 4; Fig. 2). Coastal states were more likely to report ≥1 Va, Vc, Vf, and Vm cases compared to non-coastal states (Table S3). For all species except Va, odds of reporting > 1 case were substantially higher for Gulf Coast states compared with Atlantic and Pacific Coast states (Table S3).

### Trends.

**Figure 3.**
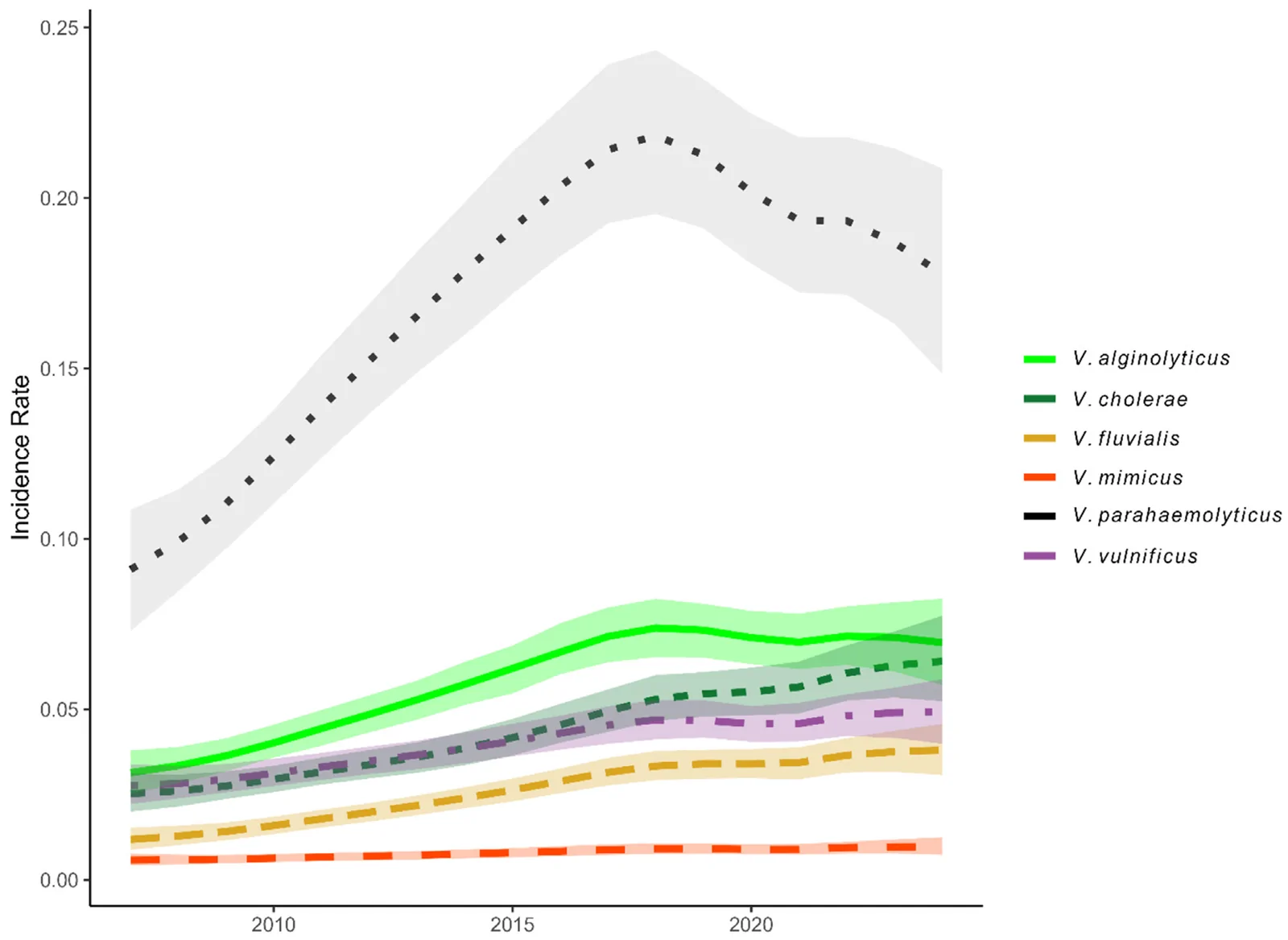
Trends in the incidence per 100,000 people of infections reported to the Cholera and Other Vibrio Illness Surveillance System (COVIS) caused by four emerging *Vibrio* species of concern, and by *V. parahaemolyticus* and *V. vulnificus* during 2007–2024 according to Bayesian spline regression.

The national incidence of all species except Vm increased over the analysis period (2007–2024; Fig 3). The incidence of Va, Vc, Vf, Vm, Vp, and Vv infection did not meaningfully decrease during the COVID-19 pandemic (2020–2021). Exclusion and inclusion of these years did not alter trend estimates, so reported results include data from 2020 and 2021.

Vf exhibited the largest relative increase in incidence with the emergence of CIDT use among the emerging species, with median incidence of infection increasing 101% (95% CrI = 64%–139%). Incidence of Va infection increased 67% (95% CrI = 45%–92%), while Vc infection increased 79% (95% CrI: 52%–109%), and Vm infection increased 38% (95% CrI = 10%–68%).

### CFRF results.

**Figure 4.**
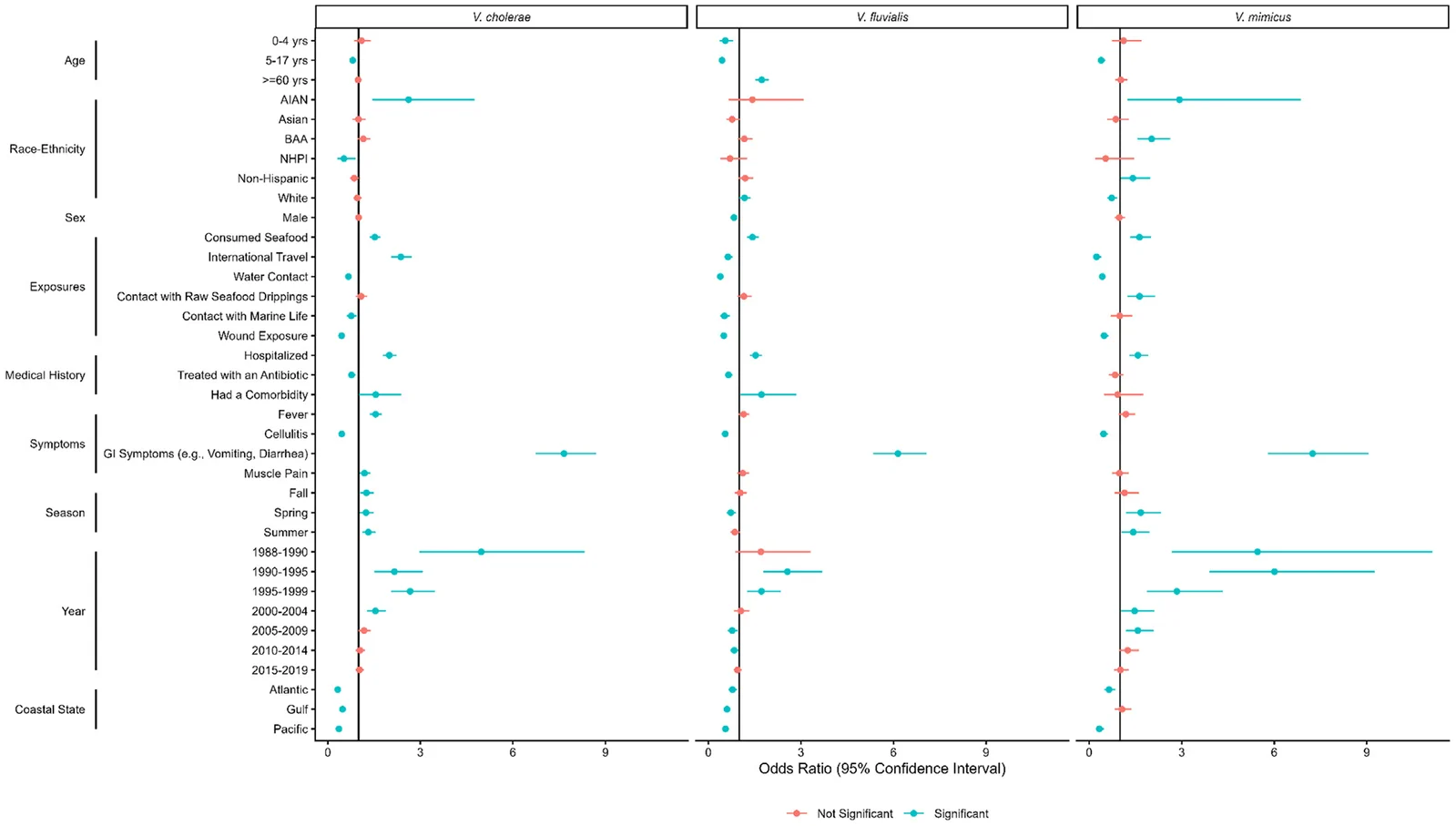
Results of the counterfactual random forests comparing odds that a person is infected with *V. cholerae*, *V. fluvialis*, and *V. mimicus* compared to *V. alginolyticus*. Odds ratios represent the odds that an ill person was infected with the given species, versus *V. alginolyticus*, among individuals who met the indicated criterion compared with those who did not. Reference levels were age 18–59 years; not belonging to the specified race or ethnic group; female sex; absence of the specified exposure; no antibiotic treatment; no reported comorbidity; absence of the specified symptom; no hospitalization; winter season; and residence in a non-coastal state. Results for patients with missing or unknown values are not shown; these categories were included as an explicit level (i.e., Not Reported) in the analysis. AIAN = American Indian or Alaska Native; BAA = Black or African American; NHPI = Native Hawaiian or Pacific Islander.

Three CFRFs were fit to identify features that distinguished emerging *Vibrio* species from each other and from the historically better characterized Vp and Vv infections. Across CFRFs, Vp and Vv behave as clinically and epidemiologically distinct (Figs. S2–S5). Va was clinically and epidemiologically distinct from Vc, Vf, and Vm. Fewer features distinguish Vc, Vf, and Vm from one another (Fig. 4). There were similar differences in odds of infection when Vp and Vv were the reference (Figs. S2–S5), so results are presented using Va as the referent.

#### Demographic characteristics:

The odds of being infected with Va, Vc, Vf, and Vm differed substantially by patient demographic characteristics. For example, compared to Va, persons with:

1. Vc (OR = 0.81, 95% Confidence Interval [95% CI] = 0.71, 0.92), Vf (OR = 0.44; 95% CI = 0.36, 0.54) and Vm (OR = 0.39; 95% CI = 0.29, 0.53) were less likely to be aged 5–17 years;
2. Vf were more likely to be ≥60 years of age (OR = 1.73; 95% CI = 1.52, 1.96) and less likely to be aged 0–4 (OR = 0.54; 95% CI = 0.36, 0.80).

Differences were observed for persons of different race and ethnicity. AIAN persons were more likely to be infected with Vc (OR = 2.62; 95% CI = 1.44, 4.76) and Vm (OR = 2.93; 95% CI = 1.25, 6.87) compared to Va, and BAA persons were more likely to be infected with Vm (OR = 2.03; 95% CI = 1.56, 2.62). NHPI persons were less likely to be infected with Vc (OR = 0.52, 95% CI = 0.31, 0.90) and Vf (OR = 0.86, 95% CI = 0.74, 0.99) than Va. We found little evidence that sex was associated with differential odds of infection by species.

#### Exposures:

Consumption of seafood was positively associated with Vc (OR = 1.52; 95% CI = 1.36, 1.71), Vf (OR = 1.43; 95% CI = 1.25, 1.63), and Vm (OR = 1.63; 95% CI = 1.33, 2.00) compared to Va. In sensitivity analyses, seafood consumption was positively associated with Va, Vc, Vf, and Vm compared to Vv, and with Vf and Vm compared to Vp (Figs. S2–S5).

Water contact was negatively associated with Vc (OR = 0.67; 95% CI = 0.60, 0.75), Vf (OR = 0.39; 95% CI = 0.34, 0.44), and Vm (OR = 0.43; 95% CI = 0.35, 0.52) compared to Va. Contact with marine life was negatively associated with Vc (OR = 0.76; 95% CI = 0.62, 0.94) and Vf (OR = 0.52; 95% CI = 0.39, 0.69) compared to Va. Wound contact with water, marine life, or raw seafood drippings was negatively associated with Vc (OR = 0.45, 95% CI = 0.40, 0.51), Vf (OR = 0.50; 95% CI = 0.43, 0.58), and Vm (OR = 0.49; 95% CI = 0.38, 0.62) compared to Va.

#### Medical history, symptoms, and treatment:

Compared to Va, cellulitis was negatively associated with Vc (OR = 0.45, 95% CI = 0.39, 0.52), Vf (OR = 0.54, 0.46, 0.64), and Vm (OR = 0.47, 95% CI = 0.35, 0.61) compared to Va. Vc (OR = 7.66; 95% CI = 6.74, 8.69), Vf (OR = 6.14; 95% CI = 5.34, 7.07), and Vm (OR = 7.24; 95% CI = 5.80, 9.05) were positively associated with gastrointestinal symptoms compared to Va.

Persons infected with Vc (OR = 1.99; 95% CI = 1.78, 2.23), Vf (OR = 1.53; 95% CI = 1.34, 1.74), and Vm (OR = 1.58; 95% CI = 1.30, 1.92) were more likely to be hospitalized than persons infected with Va. Persons infected with Vc and Vf were more likely to report data on one or more comorbidities than persons infected with Va.

## Discussion

This analysis is the first to systematically describe and compare the epidemiology of infections caused by four emerging *Vibrio* species of concern in the United States with one another and to infections caused by Vp and Vv. The findings show that infections caused by Va, Vc, Vf, and Vm have increased over time and exhibit substantial heterogeneity in epidemiology across species. Incidence varied by demographic group, season, and geography, but was consistently higher in coastal states. Species-specific patterns were evident in age distribution, reported exposures, and clinical presentation, with Va differing notably from the other emerging species. These findings highlight that emerging *Vibrio* infections represent multiple, distinct epidemiologic profiles.

### Although the four emerging *Vibrio* species of concern contribute substantially to vibriosis burden in the United States, changing diagnostic practices complicate interpretation of long-term trends.

Following designation of vibriosis as a nationally notifiable condition in 2007, the incidence of infections caused by Va, Vc, Vf, and Vm significantly increased. Notably, Va overtook Vv as the second most commonly reported cause of vibriosis in the United States (Newton et al., 2012). The largest and most sustained increases were observed for Vc and Vf. This mirrors trends reported by the 10-state Foodborne Diseases Active Surveillance Network (FoodNet) as well as earlier analyses that used COVIS data through 2012 (Jacobs Slifka et al., 2017; Newton et al., 2012). These findings indicate that illnesses caused by these species now comprise a substantial and growing component of the national vibriosis burden.

Interpretation of longer-term vibriosis trends is complicated by changes in diagnostic practices. Increased use of CIDTs, particularly syndromic panel tests, over the past decade has influenced reported incidence for several enteric pathogens (Shah et al., 2024). While incidence estimates for all four emerging *Vibrio* species increased after widespread adoption of CIDTs, rates of increase subsequently slowed or plateaued since then. Prior modeling work has suggested that removal of CIDT effects would reduce, but not eliminate, observed increases in vibriosis, and that the impact of CIDTs on vibriosis was smaller than for other enteric illnesses (Healy et al., 2024). These findings suggest that recent increases in Va, Vc, Vf, and Vm incidence reflect a combination of true epidemiologic increase and changes in detection, with CIDTs contributing to, but not driving, the observed trends.

CIDTs do not produce a bacterial isolate, which is required for species-level identification (Shah et al., 2024). There has been an overall decrease in the number of clinical labs that perform reflex culture after a positive CIDT (Ray et al., 2022), and according to the 2023 FoodNet report, vibriosis CIDT-positive reflex culture decreased from 61% during 2016–2018 to 34% in 2023, while the incidence of unspeciated vibriosis infections increased more than fourfold (Shah et al., 2024). This may lead to under-attribution of vibriosis caused by specific *Vibrio* species, which may partially explain the plateauing in incidence observed for several of the species here. While CIDTs can generate false-positive results due to *Vibrio* nucleic acid contamination of the media used to collect and preserve stool specimens (Decuir et al., 2021), the present analysis only considered confirmed vibriosis cases. In addition, COVIS does not collect information on the laboratory methods used for species identification. Laboratories may use different approaches (e.g., phenotypic testing, MALDI-TOFMS, molecular methods, or whole-genome sequencing), and changes in identification practices over time could have influenced species ascertainment. Together, this suggests that species-specific trends should be interpreted cautiously and in the context of the evolving diagnostic landscape.

### Species-specific regional patterns suggest opportunities for targeted prevention.

Across all four emerging species of concern, infections were strongly associated with residence in coastal states. This is consistent with our understanding of vibriosis; *Vibrio* naturally occur in marine and estuarine environments (Jones, 2014), and therefore, many of the primary transmission routes to humans (i.e., seafood consumption and/or contact with marine waters, marine life, or sea-food drippings) are more common in coastal states (Cody et al., 2024; Jones, 2014; Mossler, 2020). Between coastal states, spatial epidemiology of Vc, Vf, and Vm was similar but distinct from Va. This suggests that surveillance, preparedness, and prevention efforts are likely to be most effective when geographically targeted. Prioritizing surveillance capacity, clinician awareness, and situational preparedness in coastal states may improve early detection and response, particularly in the Gulf Coast and Hawaii. Aligning public health resources and improving clinician awareness of the most prevalent species, associated exposure risks, and clinical presentations in each region may further enhance effectiveness.

### Exposures, transmission patterns, and clinical presentation of Va infections differ substantively from Vc, Vf, and Vm infections.

The differences in clinical presentation between *Vibrio* species mirrored differences in reported exposure. Va infections were infrequently foodborne and often associated with water exposure, wound contact, and interaction with marine life. In comparison, Vc, Vf, and Vm infections were more frequently foodborne and often linked to seafood consumption. These findings mirror those of other studies (Alam et al., 2023; Chitov et al., 2008; Dechet et al., 2008; Dzubian et al., 2006; Kay et al., 2012). These patterns suggest that Va, like Vv, is a predominantly water-associated pathogen with some foodborne exposure. Vc, Vf, and Vm are mostly foodborne pathogens with some non-foodborne transmission, like Vp. This may reflect differences in the ecology of the various species; Vc, Vf, and Vm belong to a subset of *Vibrio* species that can live in low-salinity environments, which may influence their density in certain waters, creating species-specific exposure opportunities (Sampaio et al., 2022). However, as noted, exposure pathways were not mutually exclusive. This overlap indicates that prevention efforts should not be framed as binary but instead should incorporate both food safety and environmental exposure prevention, with emphasis tailored to the dominant transmission pathways for each species.

Consistent with two European studies (Hoefler et al., 2022; Tapia-Poza et al., 2025), Va infections more often caused SSTIs and showed a distinctive association with ear infections compared to Vc, Vf, Vm, and Vp. In contrast, Vc, Vf, and Vm infections were more commonly associated with gastrointestinal illness and systemic symptoms, such as fever and muscle pain (Tapia-Poza et al., 2025). Differences in diagnostic specimen sources were consistent with these patterns, with Va infections most often identified from wound or other non-stool specimens (Hlady & Klontz, 1996). A Spanish study showed that *Vibrio* human clinical isolates clustered by both species and clinical presentation, with Va forming a predominantly ear infection-associated clade, and Vc and Vf forming a predominantly gastrointestinal clade (Tapia-Poza et al., 2025). Clinical pattern recognition could support earlier identification and reporting of Va, which has a distinctive clinical and exposure signal that can increase clinical suspicion and prompt appropriate testing. Such syndromic awareness may complement, but not replace, laboratory-based diagnosis and species confirmation.

Although illness severity differed by species, Va, Vc, Vf, and Vm were associated with substantial morbidity, but not mortality, consistent with past epidemiologic reports (Hlady & Klontz, 1996; Hoefler et al., 2022; Newton et al., 2012). Approximately 40% of patients with Vc, Vf, and Vm infections were hospitalized or had other severe clinical symptoms (e.g., bloody diarrhea, septic shock), similar to Vp. Comparatively, this was only 13% for Va infections (noting, however, that 22% of Va infections did not have hospitalization data reported). Rates of hospitalization and severe outcomes (e.g., septic shock) were substantially lower for all four emerging species compared to Vv, concurring with past studies (Hoefler et al., 2022; Newton et al., 2012). This suggests a spectrum of illness severity, with Vv causing the most severe illnesses and Va the least. Improving clinician knowledge of the existence and potential severity of emerging *Vibrio* species may promote earlier diagnoses and improve patient outcomes.

### Demographic patterns reinforce differences in exposure and clinical presentation between species and suggest population-specific prevention strategies are needed.

Va infections were associated with younger age groups, while Vc, Vf, and Vm infections were more frequently observed among older adults. Differences in transmission and clinical presentation of Va compared to Vc, Vf, and Vm suggest that age-specific risk differs across *Vibrio* species and may reflect differences in exposure contexts or biological susceptibility. Differences by race and ethnicity were also observed across emerging species. Incidence was consistently highest across species among people identifying as NHPI, aligning with geographical data showing Hawaii as a hotspot, indicating a substantial and persistent vibriosis burden for this population. This may reflect localized public health expertise at the Hawaii Public Health Lab (personal correspondence with C. Lee). Past studies have shown that seafood consumption and water exposures vary by race-ethnicity, which may help explain some of the race-ethnicity patterns observed here, though other structural and contextual factors also likely contribute to these differences (Devine et al., 2025; U.S. Centers for Disease Control and Prevention, 2022; Sechena et al., 2003; Terry et al., 2013; Baker et al., 2020; Sechena et al., 2003). These findings suggest that public health interventions may be most effective when tailored to populations experiencing higher incidence and to the specific *Vibrio* species and clinical presentations contributing to disease burden within those populations.

## Conclusion

The four emerging *Vibrio* species considered here, Va, Vc, Vf and Vm, represent a growing burden of illness in the United States associated with substantial morbidity. Infections increased over time for all species and were consistently concentrated in coastal regions, reinforcing their emerging importance for public health and clinical practice in the United States. The four species differed by a number of factors, including geographic incidence, case demographics, exposure pathway, and clinical presentation, indicating that they should not be treated as a single epidemiologic group. These differences provide potential avenues to tailor prevention efforts to both foodborne and non-foodborne transmission in particular regions and populations at higher risk. Clinician education to support earlier identification of non-traditional vibriosis infections (such as ear infections) can further improve diagnostic accuracy and reduce patient morbidity. These findings also highlight the importance of continued infectious disease surveillance for infections caused by specific *Vibrio* species in the context of changing diagnostic practices, which requires dedicated resources for state and local public health partners.

## Supplementary Material

Supplemental Materials

## Figures and Tables

**Table 1:** Total number of illnesses (No.) and illness incidence per 100,000 persons (IR) among different populations, regions, and time periods reported to the Cholera and Other Vibrio Illness Surveillance System (COVIS) during 2007–2024 by etiologic agent^a^

	*Vibrio* species (Total No. of Illnesses and IR During 2007–2024)											
	Emerging Species of Concern								Comparison Species			
	*alginolyticus* (3,846)		*cholera* (2,105)		*fluvialis* (1,381)		*mimicus* (421)		*parahaemolyticus* (8,930)		*vulnificus* (2,632)	
	No.	IR	No.	IR	No.	IR	No.	IR	No.	IR	No.	IR
Demographics												
Age												
<1	3	0.004	17	0.024	4	0.006	3	0.004	5	0.007	1	0.001
1–4	158	0.056	66	0.023	7	0.002	8	0.003	49	0.017	7	0.002
5–17	1041	0.107	174	0.018	51	0.005	17	0.002	400	0.041	92	0.009
18–59	1777	0.055	1116	0.035	588	0.018	233	0.007	5949	0.184	951	0.029
>60	802	0.066	708	0.058	718	0.059	155	0.013	2383	0.196	1549	0.128
NR ^b^	65	–	24	–	13	–	5	–	144	–	32	–
Ethnicity												
Hispanic	441	0.043	310	0.030	123	0.012	30	0.003	944	0.093	244	0.024
Non-Hispanic	2386	0.050	1396	0.029	961	0.020	289	0.006	5487	0.123	1942	0.041
NR	1019	–	399		297	–	102	–	2139	–	446	–
Race												
AIAN ^c^	10	0.014	32	0.044	11	0.015	6	0.008	40	0.055	9	0.012
Asian	139	0.043	135	0.042	57	0.018	12	0.004	323	0.099	69	0.021
BAA ^c^	165	0.022	239	0.031	129	0.017	83	0.011	528	0.069	148	0.019
NHPI ^c^	65	0.545	7	0.059	9	0.075	2	0.017	61	0.511	28	0.235
White	2548	0.057	1354	0.031	960	0.022	261	0.006	6123	0.138	2095	0.047
Multiple Races	6	0.004	3	0.002	1	0.001	0	0	9	0.006	1	0.001
Another Race	119	–	53	–	33	–	9	–	229	–	44	–
NR	794	–	282	–	181	–	48	–	1617	–	238	–
Sex												
Female	1264	0.043	811	0.028	578	0.020	161	0.006	3031	0.104	449	0.015
Male	2546	0.090	1276	0.045	785	0.028	257	0.009	5799	0.204	2148	0.076
NR	36	–	18	–	18	–	3	–	100	–	35	–
Geography												
Coastal												
Atlantic	1310	0.087	432	0.029	512	0.034	111	0.007	2998	0.199	755	0.05
Gulf Coast	1335	0.123	693	0.064	430	0.040	208	0.019	1299	0.120	1534	0.141
Pacific	810	0.087	250	0.027	153	0.016	25	0.003	3001	0.322	120	0.013
Non-coastal	391	0.017	730	0.033	286	0.013	77	0.003	1632	0.073	223	0.01
Timing of Illness												
Season												
Fall	1063	0.018	502	0.009	400	0.007	79	0.001	2142	0.009	786	0.007
Spring	654	0.011	435	0.008	233	0.004	108	0.002	974	0.006	396	0.040
Summer	1702	0.029	941	0.016	594	0.010	196	0.003	5346	0.025	1354	0.012
Winter	427	0.007	227	0.004	154	0.003	38	0.001	468	0.002	96	0.001
Year^d^												
1988–1989	15	–	65	–	12	–	17	–	66	–	69	–
1990–1994	40	–	106	–	55	–	42	–	157	–	208	–
1995–1999	62	–	195	0.015	82	–	41	–	678	–	364	–
2000–2004	236	–	261	–	131	–	39	–	884	–	487	–
2005–2006	487	–	337	–	150	–	83	–	1521	–	521	–
2007–2009	336	0.037	213	0.023	89	0.010	61	0.007	898	0.10	291	0.032
2010–2014	934	0.060	482	0.031	278	0.018	109	0.007	2408	0.153	634	0.04
2015–2019	1320	0.081	723	0.045	496	0.031	128	0.008	3228	0.199	853	0.053
2020–2024	1256	0.075	687	0.041	518	0.031	123	0.007	2396	0.144	854	0.051

a While vibriosis case data were transmitted to CDC during 1988–2006, vibriosis did not become a nationally notifiable condition until 2007. From 1988 through 2007, the number of reporting states increased steadily; however, because the COVIS catchment area was fluid during this period, it is not feasible to calculate incidence prior to 2007 due to the absence of a clearly defined denominator.

b NR=Not Reported.

c AIAN = American Indian or Alaska Native; BAA = Black or African American; NHPI=Native Hawaiian or Other Pacific Islander.

d Year intervals preserve 5-year periods where possible; however, shorter intervals were used to reflect key changes in surveillance or diagnostic practices (e.g., COVIS becoming national in 2007).

**Table 2:** Number (No.) and percent of cases reporting specific clinical outcomes, symptoms, and related information reported to the Cholera and Other Vibrio Illness Surveillance System (COVIS) during 1988–2024 by etiologic agent^a,b^

	*Vibrio* species (Total No. of Illnesses During 1988–2024)											
	Emerging Species of Concerns								Comparison Specie			
	*alginolyticus* (No = 4350)		*cholerae* (No = 2856)		*fluvialis* (No = 1722)		*mimicus* (No = 582)		*parahaemolyticus* (No = 11338)		*vulnificus* (No = 3990)	
	No.	%	No.	%	No.	%	No.	%	No.	%	No.	%
Clinical Outcomes												
Did the patient die?												
No	3672	84	2447	86	1468	85	522	90	9802	86	2599	65
Not Reported	636	15	299	10	213	12	51	9	1433	13	396	10
Yes	42	1	110	4	41	2	9	2	103	1	995	25
Was the patient hospitalized?												
No	2847	65	1426	50	886	51	302	52	8014	71	420	11
Not Reported	948	22	244	9	197	11	60	10	1111	10	212	5
Yes	555	13	1186	42	639	37	220	38	2213	20	3358	84
Clinical Symptoms												
Abdominal Cramping												
No	2396	55	716	25	490	28	140	24	2315	20	1948	49
Not Reported	1757	40	596	21	411	24	120	21	2423	21	1317	33
Yes	197	5	1544	54	821	48	322	55	6600	58	725	18
Blood in Stools												
No	2649	61	1864	65	1073	62	361	62	7468	66	2358	59
Not Reported	1659	38	720	25	488	28	156	27	2649	23	1500	38
Yes	42	1	272	10	161	9	65	11	1221	11	132	3
Bullae												
No	2372	55	1978	69	1144	66	397	68	8265	73	1500	38
Not Reported	1738	40	814	29	553	32	178	31	2887	25	1454	36
Yes	240	6	64	2	25	1	7	1	186	2	1036	26
Cellulitis												
No	1887	43	1902	67	1100	64	387	66	7859	69	798	20
Not Reported	1381	32	732	26	486	28	154	26	2434	21	851	21
Yes	1082	25	222	8	136	8	41	7	1045	9	2341	59
Diarrhea												
No	2607	60	477	17	293	17	88	15	1410	12	1957	49
Not Reported	1499	34	346	12	270	16	86	15	1277	11	1108	28
Yes	244	6	2033	71	1159	67	408	70	8651	76	925	23
Ear Infection and Earache^c^												
Not Reported	3335	77	2791	98	1703	99	576	99	11198	99	3970	99
Yes	1015	23	65	2	19	1	6	1	140	1	20	1
Fever												
No	2430	56	1362	48	908	53	284	49	6210	55	930	23
Not Reported	1513	35	628	22	439	25	143	25	2561	23	915	23
Yes	407	9	866	30	375	22	155	27	2567	23	2145	54
Muscle Pain												
No	2469	57	1485	52	935	54	318	55	6289	55	1371	34
Not Reported	1646	38	779	27	518	30	176	30	2599	23	1418	36
Yes	235	5	592	21	269	16	88	15	2450	22	1201	30
Septic Shock												
No	2624	60	1926	67	1157	67	393	68	8311	73	1569	39
Not Reported	1681	39	794	28	524	30	168	29	2831	25	1298	33
Yes	45	1	136	5	41	2	21	4	196	2	1123	28
Sequelae												
No	2817	65	1931	68	1141	66	395	68	7739	68	1899	48
Not Reported	1375	32	855	30	536	31	173	30	3387	30	1363	34
Yes	158	4	70	2	45	3	14	2	212	2	728	18
Vomiting												
No	2689	62	1391	49	904	52	258	44	6209	55	1936	49
Not Reported	1503	35	482	17	383	22	121	21	1823	16	1060	27
Yes	158	4	983	34	435	25	203	35	3306	29	994	25
Diagnostics												
CIDT	93	2	525	18	235	14	67	12	1329	12	61	2
Culture	4257	98	2331	82	1485	86	515	88	10007	88	3929	98
Not Reported	0	0	0	0	2	<1	0	0	2	<1	0	0
Specimen Source^d^												
Was a blood sample tested?	125	3	415	15	99	6	34	6	306	3	2612	65
Was a stool sample tested?	146	3	1943	68	1247	72	418	72	9012	79	199	5
Was a wound sample tested?	2059	47	189	7	184	11	50	9	1363	12	1272	32
Was a different sample type collected and tested? ^e^	2052	47	348	12	210	12	85	15	657	6	359	9
Was an antibiotic prescribed?												
No	260	6	431	15	297	17	87	15	2705	24	146	4
Not Reported	809	19	487	17	371	22	105	18	2198	19	529	13
Yes	3281	75	1938	68	1054	61	390	67	6435	57	3315	83

a Of persons with a *V. cholerae* infection, 253 developed cholera, and 2603 developed vibriosis. All persons who developed cholera were infected with toxigenic Vc. Forty-six people infected with toxigenic Vc developed vibriosis.

b Illness duration varied by pathogen. Median illness duration was 10 days for *V. alginolyticus* (25th percentile [Q1]: 5; 75th percentile [Q3]: 21) and *V. cholerae* (Q1: 4; Q3: 7), and 7 days for *V. fluvialis* (Q1: 5; Q3: 14), *V. mimicus* (Q1: 4; Q3: 10), *V. parahaemolyticus* (Q1: 4; Q3: 10), and *V. vulnificus* (Q1: 3; Q3: 13). Interquartile ranges spanned 3–21 days, with the greatest variability observed for *V. alginolyticus* and *V. fluvialis*.

c To identify cases with ear infections and ear-related symptoms, natural language processing of a free-text response symptoms field was performed. The ear infection and ear-related symptom data represent presence only and can have values of ‘yes’ or ‘not reported’.

d Multiple specimen types could be collected from the same patient. These variables indicate whether a given specimen type was collected. Each count represents a unique infection associated with that specimen type; therefore, counts across specimen types are not mutually exclusive and may sum to more than the total number of infections.

e Other specimen types collected included cerebrospinal fluid (Va = 1; Vf = 2; Vv = 2); corneal or ocular (Va = 7; Vv = 5); ear or otic (Va = 423; Vc = 33; Vf = 3; Vm = 2; Vp = 41; Vv = 1); biliary, gallbladder, or hepatic (Va = 1; Vc = 2; Vf = 11; Vm = 2; Vp = 3; Vv = 3); intraabdominal or peritoneal (Va = 8; Vc = 9; Vf = 2; Vm = 2; Vp = 8; Vv = 1); joint or synovial (Va = 1; Vc = 1; Vm = 2; Vp = 1; Vv = 1); respiratory (Va = 75; Vc = 8; Vf = 14; Vp = 15; Vv = 1); and urinary, urogenital, or catheter-associated specimens (Va = 5; Vc = 14; Vf = 9; Vm = 6; Vp = 9; Vv = 5), among others.

**Table 3:** Number (No.) and percent of cases reporting foodborne and non-foodborne exposures by etiologic agent reported to the Cholera and Other Vibrio Illness Surveillance System (COVIS) during 1988–2024

	*Vibrio* species (Total No. of Illnesses During 1988–2024)											
	Emerging Species of Concern								Comparison Species			
	*alginolyticus* (4350)		*cholerae* (2856)		*fluvialis* (1722)		*mimicus* (582)		*parahaemolyticus* (11338)		*vulnificus* (3990)	
	No.	%	No.	%	No.	%	No.	%	No.	%	No.	%
Transmission Pathway												
Foodborne	132	3	1934	68	1238	72	414	71	8941	79	765	19
Likely Foodborne	138	3	60	2	64	4	22	4	200	2	146	4
Likely Not Foodborne	524	12	233	8	66	4	21	4	292	3	135	3
Not Foodborne	2949	68	265	9	179	10	64	11	1533	14	1966	49
Not Reported	607	14	364	13	175	10	51	10	372	3	978	25
Did the patient have any water or contact with sea life exposure?												
No	679	16	1233	43	878	51	280	48	5239	46	741	19
Not Reported	920	21	588	21	454	26	137	24	2318	20	637	16
Yes	2751	63	1035	36	390	23	165	28	3781	33	2612	65
Did the patient consume… ^a^												
any seafood?												
No	1789	41	658	23	361	22	103	18	1165	10	1087	27
Not Reported	1739	40	632	22	440	26	132	23	1841	16	1130	28
Yes	822	10	1566	55	921	53	347	60	8332	73	1773	44
any raw seafood?^b^												
No	2278	52	1286	45	669	39	242	42	2741	24	1669	42
Not Reported	1881	43	822	29	545	32	153	26	2576	23	1300	33
Yes	191	4	748	26	508	30	187	32	6021	53	1021	26
any bivalves?												
No	2254	52	1260	44	641	37	208	36	2417	21	1556	39
Not Reported	1853	43	813	28	517	30	168	29	2262	20	1265	32
Yes	243	6	783	27	564	33	206	35	6659	59	1169	29
any raw bivalves?												
No	2367	54	1389	49	717	42	232	40	2981	26	1685	42
Not Reported	1883	43	863	30	560	33	176	30	2730	24	1332	33
Yes	100	2	604	21	445	26	174	30	5627	50	973	24
clams?												
No	2348	54	1667	58	953	55	313	54	6194	55	2079	52
Not Reported	1899	44	1019	36	647	38	241	41	3708	33	1776	45
Yes	103	2	170	6	122	7	28	5	1436	13	135	3
crabs?												
No	2282	52	1518	53	875	51	272	47	5733	51	1880	47
Not Reported	1927	44	1019	36	652	38	234	40	3857	34	1732	43
Yes	141	3	319	11	195	11	76	13	1748	15	378	9
crawfish?												
No	2242	52	1492	52	857	50	269	46	5598	49	1850	46
Not Reported	1989	46	1116	39	709	41	251	43	4310	38	1856	47
Yes	119	3	248	9	156	9	62	11	1430	13	284	7
fish?												
No	1908	44	1188	42	714	41	250	43	4825	43	1708	43
Not Reported	1952	45	1007	35	667	39	226	39	3888	34	1786	45
Yes	490	11	661	23	341	20	106	18	2625	23	496	12
oysters?												
No	2313	53	1334	47	673	39	215	37	2757	24	1577	40
Not Reported	1894	44	864	30	559	32	175	30	2542	22	1315	33
Yes	143	3	658	23	490	28	192	33	6039	53	1098	28
raw oysters?												
No	2356	54	1396	49	710	41	231	40	3076	27	1671	42
Not Reported	1910	44	902	32	597	35	180	31	2981	26	1379	35
Yes	84	2	558	20	415	24	171	29	5281	47	940	24
shrimp?												
No	1830	48	899	43	593	43	184	44	4117	46	1243	47
Not Reported	1692	44	680	32	504	36	143	34	2912	33	1074	41
Yes	324	8	526	25	284	21	94	22	1901	21	315	12
Did the patient report travel…												
anywhere?												
No	2017	52	987	47	781	57	256	61	4807	54	1799	68
Not Reported	890	20	284	13	283	20	77	18	1474	17	429	16
Yes	939	24	834	40	317	23	88	21	2649	30	404	15
internationally?												
No	3035	70	1654	58	1232	72	448	77	8536	75	3178	80
Not Reported	1030	24	478	17	371	22	120	21	1999	18	756	19
Yes	7	7	724	25	119	7	14	2	803	7	56	1
Did the patient have…												
contact with any marine life including a bite or sting?												
No	2251	52	1805	63	1072	62	374	64	6952	61	1784	45
Not Reported	1751	40	924	32	604	35	180	31	3760	33	1546	39
Yes	348	8	127	4	46	3	28	5	626	6	660	17
a wound exposed to water?												
No	1325	30	1595	56	944	55	308	53	6065	53	896	22
Not Reported	1405	32	1024	36	651	38	234	40	3842	34	1217	31
Yes	1620	37	237	8	127	7	40	7	1431	13	1877	47
contact with any bodies of water? ^c^											
No	590	15	1032	49	776	56	245	58	4623	52	630	24
Not Reported	870	23	449	21	364	26	99	24	1956	22	429	16
Yes	2386	62	624	30	241	17	77	18	2351	26	1573	60
contact with drippings from raw seafood?												
No	2425	56	1661	58	949	55	306	53	6330	56	1458	37
Not Reported	1652	38	869	30	594	34	178	31	3431	30	1259	32
Yes	273	6	326	11	179	10	98	17	1577	14	1273	32
Was patient exposed through their occupation?^d^											
No	1223	28	1358	48	878	51	276	47	5200	46	1236	31
Not Reported	3097	71	878	52	838	49	304	52	6105	54	2710	68
Yes	30	1	6	<1	6	<1	2	<1	33	<1	44	1

a Relatively few persons reported consuming lobster, mussels, scallops, or other shellfish. Across species, reported consumption ranged from 1% to 6%: lobster (115 [3%] *V. alginolyticus*, 125 [4%] *V. cholerae*, 83 [5%] *V. fluvialis*, 18 [3%] *V. mimicus*, 700 [6%] *V. parahaemolyticus*, 59 [1%] *V. vulnificus*), mussels (45 [1%], 93 [3%], 58 [3%], 15 [3%], 571 [5%], 53 [1%]), scallops (27 [1%], 31 [1%], 25 [2%], 3 [1%], 179 [2%], 8 [<1%]), and other shellfish (36 [1%], 75 [4%], 42 [3%], 8 [2%], 397 [4%], 31 [1%]), respectively.

b Less than 1% of ill persons reported consuming raw lobster, and ≤1% reported consuming raw crabs, crawfish, mussels, or shrimp across all species considered. Reported raw clam consumption ranged from <1% to 5% (20 [<1%] *V. alginolyticus*, 62 [2%] *V. cholerae*, 48 [3%] *V. fluvialis*, 10 [2%] *V. mimicus*, 571 [5%] *V. parahaemolyticus*, 56 [1%] *V. vulnificus*), while raw fish consumption ranged from 1% to 6% (80 [2%], 113 [4%], 79 [5%], 14 [2%], 693 [6%], and 52 [1%], respectively).

c Contact with different water types was partially derived from fixed-response fields and natural language processing of free-text data. Saltwater contact was most common, particularly among *V. alginolyticus* (2,138 [49%]) and *V. vulnificus* (1,183 [30%]) infections, and ranged from 9% to 16% for other species. Freshwater and brackish water exposures were less frequent (freshwater: 2%–8%; brackish: 2%–16%), with brackish water most commonly reported among *V. vulnificus*. Floodwater and treated recreational water exposures were rare across all species (≤1%).

d Data on patients’ occupations were generated solely using natural language processing of free-text data. Crabbing or waterman occupations were infrequently reported, including 41 (1%) of persons with *V. alginolyticus*, 8 (<1%) with *V. cholerae*, 3 (<1%) with *V. fluvialis*, 5 (1%) with *V. mimicus*, 58 (1%) with *V. parahaemolyticus*, and 108 (3%) with *V. vulnificus* infection. This infrequency may reflect the free-response nature of the data element.

**Table 4:** Probability that states in different coastal regions reported ≥1 *Vibrio* spp. infection, or ≥1 infection caused by specific *Vibrio* species reported to the Cholera and Other Vibrio Illness Surveillance System (COVIS) during 1988–2024 for states in different coastal regions

	Probability of a State Reporting ≥1 Case in a Given Year		
	Mean	95% Credible Interval	
		Lower	Upper
*Vibrio* spp.			
Noncoastal	0.22	0.17	0.29
Atlantic	0.51	0.40	0.62
Gulf Coast	0.90	0.82	0.95
Pacific	0.55	0.37	0.72
Emerging Species of Concern			
*V. alginolyticus*			
Noncoastal	0.09	0.05	0.16
Atlantic	0.59	0.37	0.77
Gulf Coast	0.72	0.42	0.91
Pacific	0.69	0.40	0.90
*V. cholera*			
Noncoastal	0.37	0.26	0.49
Atlantic	0.43	0.28	0.59
Gulf Coast	0.92	0.81	0.98
Pacific	0.61	0.37	0.82
*V. fluvialis*			
Noncoastal	0.11	0.06	0.16
Atlantic	0.44	0.28	0.61
Gulf Coast	0.80	0.61	0.92
Pacific	0.28	0.11	0.51
*V. mimicus*			
Noncoastal	0.03	0.02	0.06
Atlantic	0.10	0.05	0.17
Gulf Coast	0.60	0.38	0.81
Pacific	0.08	0.03	0.20
Comparison Species			
*V. parahaemolyticus*			
Noncoastal	0.46	0.30	0.61
Atlantic	0.89	0.79	0.96
Gulf Coast	1.00	0.99	1.00
Pacific	0.97	0.90	0.99
*V. vulnificus*			
Noncoastal	0.17	0.09	0.26
Atlantic	0.54	0.35	0.72
Gulf Coast	0.99	0.96	1.00
Pacific	0.43	0.16	0.73

## Data Availability

The data underlying this article cannot be shared publicly due to data privacy laws. De-identified data may be available on request by contacting COVISResponse@cdc.gov, per data sharing policies.

## Appendix A. Supplementary material

Supplementary material to this article can be found online at https://doi.org/10.1016/j.jfp.2026.100891.
