# Supplementary material for: Epidemiology of Infections Caused by Four Emerging Vibrio Species of Concern Reported to the Cholera and Other Vibrio Illness Surveillance System (COVIS), United States, 1988–2024
Source: J Food Prot. Author manuscript; Available in PMC 2026 Sep 15. (PMC13572979; doi:10.1016/j.jfp.2026.100891)
Supplement: Supplementary file 1 [file NIHMS2206465-supplement-Supplemental_Materials.docx]

Title: Epidemiology of infections caused by four emerging *Vibrio* species of concern reported to the Cholera and Other Vibrio Illness Surveillance system (COVIS), United States, 1988–2024

Authors: Daniel L. Weller ^a^, Marisa Hast ^a^, Robin Pendley Louis ^a^, Christine C. Lee ^a^, and Michael Hughes ^a^

Supplemental Material

Fig S1: Total number of infections caused by each *Vibrio* species reported to the Cholera and Other Vibrio Illness Surveillance System (COVIS) during 1988–2024.

Figure S2: Results of the counterfactual random forests comparing odds that a person is infected with *V. alginolyticus* compared to *V. parahaemolyticus* and *V. vulnificus*. Odds ratios represent the odds that an ill person was infected with *V. alginolyticus*, versus the reference species*,* among individuals who met the indicated criterion compared with those who did not. Reference levels were age 18–59 years; not belonging to the specified race or ethnic group; female sex; absence of the specified exposure; no antibiotic treatment; no reported comorbidity; absence of the specified symptom; no hospitalization; winter season; and residence in a non-coastal state. Year of infection and region were controlled for in the model but results are not shown. Results for patients with missing or unknown values are not shown; these categories were included as an explicit level (i.e., Not Reported) in the analysis. AIAN = American Indian or Alaska Native; BAA = Black or African American.

Figure S3: Results of the counterfactual random forests comparing odds that a person is infected with *V. cholerae* compared to *V. alginolyticus, V. parahaemolyticus* and *V. vulnificus*. Odds ratios represent the odds that an ill person was infected with *V. cholerae*, versus the reference species*,* among individuals who met the indicated criterion compared with those who did not. Reference levels were age 18–59 years; not belonging to the specified race or ethnic group; female sex; absence of the specified exposure; no antibiotic treatment; no reported comorbidity; absence of the specified symptom; no hospitalization; winter season; and residence in a non-coastal state. Year of infection and region were controlled for in the model but results are not shown. Results for patients with missing or unknown values are not shown; these categories were included as an explicit level (i.e., Not Reported) in the analysis. AIAN = American Indian or Alaska Native; BAA = Black or African American.

Figure S4: Results of the counterfactual random forests comparing odds that a person is infected with *V. fluvialis* compared to *V. alginolyticus, V. parahaemolyticus* and *V. vulnificus*. Odds ratios represent the odds that an ill person was infected with *V. fluvialis*, versus the reference species*,* among individuals who met the indicated criterion compared with those who did not. Reference levels were age 18–59 years; not belonging to the specified race or ethnic group; female sex; absence of the specified exposure; no antibiotic treatment; no reported comorbidity; absence of the specified symptom; no hospitalization; winter season; and residence in a non-coastal state. Year of infection and region were controlled for in the model but results are not shown. Results for patients with missing or unknown values are not shown; these categories were included as an explicit level (i.e., Not Reported) in the analysis. AIAN = American Indian or Alaska Native; BAA = Black or African American.

Figure S5: Results of the counterfactual random forests comparing odds that a person is infected with *V. mimicus* compared to *V. alginolyticus, V. parahaemolyticus* and *V. vulnificus*. Odds ratios represent the odds that an ill person was infected with *V. mimicus*, versus the reference species*,* among individuals who met the indicated criterion compared with those who did not. Reference levels were age 18–59 years; not belonging to the specified race or ethnic group; female sex; absence of the specified exposure; no antibiotic treatment; no reported comorbidity; absence of the specified symptom; no hospitalization; winter season; and residence in a non-coastal state. Year of infection and region were controlled for in the model but results are not shown. Results for patients with missing or unknown values are not shown; these categories were included as an explicit level (i.e., Not Reported) in the analysis. AIAN = American Indian or Alaska Native; BAA = Black or African American.

Table S1: Prior distributions used in Bayesian generalized additive mixed models comparing the odds that a state reported ≥1 *Vibrio* infection to the Cholera and Other Vibrio Illness Surveillance System (COVIS) during 1988–2024, by etiologic agent, coastal status and region. ^a^

| Species | Intercept | Fixed Effects | SD | | |
| --- | --- | --- | --- | --- | --- |
|  |  |  | Spline for Year | Spline for log_10_(population) | Random Intercept |
| *V. alginolyticus* | Normal(−1.9, 3.10) | Normal(0, 2.02) | Exponential(1.41) | Exponential(0.92) | Normal(0, 2.03) |
| *V. cholerae* | Normal(−2.1, 2.9) | Normal(0, 2.18) | Exponential(1.73) | Exponential(1.02) | Normal(0, 2.8) |
| *V. fluvialis* | Normal(−2.1, 2.9) | Normal(0, 2.58) | Exponential(1.8) | Exponential(0.87) | Normal(0, 2.52) |
| *V. mimicus* | Normal(−2.7, 2.31) | Normal(0, 3.27) | Exponential(2.7) | Exponential(0.7) | Normal(0, 3.41) |
| *V. parahaemolyticus* | Normal(−1.8, 3.2) | Normal(0, 2.8) | Exponential(1.33) | Exponential(1.13) | Normal(0, 2.5) |
| *V. vulnificus* | Normal(−1.6, 3.4) | Normal(0, 4.6) | Exponential(2.93) | Exponential(0.86) | Normal(0, 3.95) |

^a^ Intercept and fixed-effect priors are specified on the logit scale. Exponential priors apply to the standard deviations of the spline coefficients. Normal priors on random-effect standard deviations are implicitly half-normal due to positivity constraints.

Table S2: Number (No.) and percent of cases reporting specific comorbidities reported to the Cholera and Other Vibrio Illness Surveillance System (COVIS) during 1988–2024 by etiologic agent.

|  |  |  | *Vibrio* species (Total No. of Illnesses During 1988-2024) | | | | | | | | | | | | | | | | |
| --- | --- | --- | --- | --- | --- | --- | --- | --- | --- | --- | --- | --- | --- | --- | --- | --- | --- | --- | --- |
|  |  |  | Emerging Species of Concern | | | | | | | | | | |  | Comparison Species | | | | |
|  |  |  | *alginolyticus* (4350) | |  | *cholerae* (2856) | |  | *fluvialis* (1722) | |  | *mimicus* (582) | |  | *parahaemolyticus* (11338) | |  | *vulnificus* (3990) | |
|  |  |  | No. | % |  | No. | % |  | No. | % |  | No. | % |  | No. | % |  | No. | % |
| Comorbidities | | | | | | | | | | | | | | | | | | | |
|  | Cancer | | | | | | | | | | | | | | | | | | |
|  |  | No | 2964 | 68 |  | 1986 | 70 |  | 1065 | 62 |  | 416 | 71 |  | 8445 | 74 |  | 2452 | 61 |
|  |  | Not Reported | 1230 | 28 |  | 662 | 23 |  | 463 | 27 |  | 130 | 22 |  | 2448 | 22 |  | 1074 | 27 |
|  |  | Yes | 156 | 4 |  | 208 | 7 |  | 194 | 11 |  | 36 | 6 |  | 445 | 2 |  | 464 | 12 |
|  | Gastric Bypass Surgery | | | | | | | | | | | | | | | | | | |
|  |  | No | 3031 | 70 |  | 1970 | 69 |  | 1095 | 64 |  | 411 | 71 |  | 8461 | 75 |  | 2615 | 66 |
|  |  | Not Reported | 1264 | 29 |  | 661 | 23 |  | 451 | 26 |  | 129 | 22 |  | 2469 | 22 |  | 1151 | 29 |
|  |  | Yes | 55 | 1 |  | 225 | 8 |  | 176 | 10 |  | 42 | 7 |  | 408 | 4 |  | 224 | 6 |
|  | Heart Disease | | | | | | | | | | | | | | | | | | |
|  |  | No | 2857 | 66 |  | 1846 | 65 |  | 975 | 57 |  | 374 | 64 |  | 8110 | 72 |  | 2011 | 50 |
|  |  | Not Reported | 1200 | 28 |  | 635 | 22 |  | 433 | 25 |  | 125 | 21 |  | 2380 | 21 |  | 937 | 23 |
|  |  | Yes | 293 | 7 |  | 375 | 13 |  | 314 | 18 |  | 83 | 14 |  | 848 | 7 |  | 1042 | 26 |
|  | Heart Failure | | | | | | | | | | | | | | | | | | |
|  |  | No | 227 | 5 |  | 215 | 8 |  | 165 | 10 |  | 40 | 7 |  | 785 | 7 |  | 436 | 11 |
|  |  | Not Reported | 4042 | 93 |  | 2555 | 89 |  | 1474 | 86 |  | 525 | 90 |  | 10410 | 92 |  | 3307 | 83 |
|  |  | Yes | 81 | 2 |  | 86 | 3 |  | 83 | 5 |  | 17 | 3 |  | 143 | 1 |  | 247 | 6 |
|  | Hematologic Disease | | | | | | | | | | | | | | | | | | |
|  |  | No | 3033 | 70 |  | 2060 | 72 |  | 1172 | 68 |  | 431 | 74 |  | 8627 | 76 |  | 2564 | 64 |
|  |  | Not Reported | 1258 | 29 |  | 712 | 25 |  | 490 | 28 |  | 140 | 24 |  | 2525 | 22 |  | 1175 | 29 |
|  |  | Yes | 59 | 1 |  | 84 | 3 |  | 60 | 3 |  | 11 | 2 |  | 186 | 2 |  | 251 | 6 |
|  | Immunosuppressive Condition | | | | | | | | | | | | | | | | | | |
|  |  | No | 2989 | 69 |  | 2015 | 71 |  | 1125 | 65 |  | 422 | 73 |  | 8460 | 75 |  | 2505 | 63 |
|  |  | Not Reported | 1262 | 29 |  | 701 | 25 |  | 500 | 29 |  | 130 | 22 |  | 2490 | 22 |  | 1182 | 30 |
|  |  | Yes | 99 | 2 |  | 140 | 5 |  | 97 | 6 |  | 30 | 5 |  | 388 | 3 |  | 303 | 8 |
|  | Immunosuppressive Therapy | | | | | | | | | | | | | | | | | | |
|  |  | No | 2777 | 64 |  | 1875 | 66 |  | 1029 | 60 |  | 378 | 65 |  | 7857 | 69 |  | 2441 | 61 |
|  |  | Not Reported | 1516 | 35 |  | 897 | 31 |  | 624 | 36 |  | 194 | 33 |  | 3240 | 29 |  | 1400 | 35 |
|  |  | Yes | 57 | 1 |  | 84 | 3 |  | 69 | 4 |  | 10 | 2 |  | 241 | 2 |  | 149 | 4 |
|  | Kidney Disease | | | | | | | | | | | | | | | | | | |
|  |  | No | 2997 | 69 |  | 2012 | 70 |  | 1118 | 65 |  | 407 | 70 |  | 8498 | 75 |  | 2430 | 61 |
|  |  | Not Reported | 1253 | 29 |  | 674 | 24 |  | 444 | 26 |  | 134 | 23 |  | 2499 | 22 |  | 1087 | 27 |
|  |  | Yes | 100 | 2 |  | 170 | 6 |  | 160 | 9 |  | 41 | 3 |  | 341 | 3 |  | 473 | 12 |
|  | Liver Disease | | | | | | | | | | | | | | | | | | |
|  |  | No | 3023 | 69 |  | 1993 | 70 |  | 1186 | 69 |  | 426 | 73 |  | 8580 | 76 |  | 2034 | 51 |
|  |  | Not Reported | 1250 | 29 |  | 648 | 23 |  | 459 | 27 |  | 136 | 23 |  | 2458 | 22 |  | 830 | 21 |
|  |  | Yes | 77 | 2 |  | 215 | 8 |  | 77 | 4 |  | 20 | 3 |  | 300 | 3 |  | 116 | 28 |
|  | Peptic Ulcer | | | | | | | | | | | | | | | | | | |
|  |  | No | 3013 | 69 |  | 2007 | 70 |  | 1137 | 66 |  | 414 | 71 |  | 8422 | 74 |  | 2579 | 65 |
|  |  | Not Reported | 1310 | 30 |  | 746 | 26 |  | 509 | 30 |  | 150 | 26 |  | 2662 | 23 |  | 1242 | 31 |
|  |  | Yes | 27 | 1 |  | 103 | 4 |  | 76 | 4 |  | 18 | 3 |  | 254 | 2 |  | 169 | 4 |
| Health Behaviors | | | | | | | | | | | | | | | | | | | |
|  | Took Antacid or Ulcer Medication | | | | | | | | | | | | | | | | | | |
|  |  | No | 1166 | 27 |  | 623 | 22 |  | 376 | 22 |  | 96 | 16 |  | 2803 | 25 |  | 652 | 16 |
|  |  | Not Reported | 3103 | 71 |  | 2040 | 71 |  | 1194 | 69 |  | 455 | 78 |  | 7787 | 69 |  | 3165 | 79 |
|  |  | Yes | 81 | 2 |  | 193 | 7 |  | 152 | 9 |  | 31 | 5 |  | 748 | 7 |  | 173 | 4 |

Table S3: Odds ratios and risk difference of reporting ≥ 1 *Vibrio* spp. infection, or ≥1 infection caused by specific *Vibrio* species during 1988–2024 for states in different coastal regions.

| Species | | Comparison | Odds Ratio | | |  | Risk Difference | | |
| --- | --- | --- | --- | --- | --- | --- | --- | --- | --- |
|  |  |  | Mean | 95% Credible Interval | |  | Mean  Mean | 95% Credible Interval |  |
|  |  |  |  | Lower | Upper |  |  | Lower | Upper |
| *Vibrio* spp. | | | | | | | | | |
|  |  | Coastal (Ref=Non-Coastal) | 5.44 | 3.43 | 8.05 |  | *0.38* | 0.28 | 0.46 |
|  |  | Atlantic Coast (Ref=Non-Coastal) | 3.81 | 2.21 | 6.05 |  | 0.29 | 0.17 | 0.40 |
|  |  | Gulf Coast (Ref=Non-Coastal) | 36.63 | 15.48 | 73.74 |  | *0.68* | 0.58 | 0.75 |
|  |  | Pacific Coast (Ref=Non-Coastal) | 4.67 | 2.02 | 9.18 |  | *0.33* | 0.15 | 0.50 |
|  |  | Gulf Coast (Ref=Atlantic Coast) | 9.99 | 4.13 | 20.43 |  | *0.39* | 0.27 | 0.51 |
|  |  | Pacific Coast (Ref=Atlantic Coast) | 1.28 | 0.54 | 2.69 |  | *0.04* | -0.15 | 0.23 |
|  |  | Pacific Coast (Ref=Gulf Coast) | 0.15 | 0.05 | 0.35 |  | *-0.35* | -0.53 | -0.17 |
| Emerging Species of Concern | | | | | | | | | |
|  | *V. alginolyticus* | | | | | | | | |
|  |  | Coastal (Ref=Non-Coastal) | 20.12 | 8.46 | 41.14 |  | 0.55 | 0.38 | 0.69 |
|  |  | Atlantic Coast (Ref=Non-Coastal) | 16.93 | 5.83 | 39.31 |  | 0.50 | 0.29 | 0.68 |
|  |  | Gulf Coast (Ref=Non-Coastal) | 36.91 | 7.16 | 115.38 |  | 0.63 | 0.33 | 0.82 |
|  |  | Pacific Coast (Ref=Non-Coastal) | 32.05 | 6.48 | 98.25 |  | 0.60 | 0.31 | 0.81 |
|  |  | Gulf Coast (Ref=Atlantic Coast) | 2.49 | 0.45 | 8.05 |  | 0.13 | -0.19 | 0.39 |
|  |  | Pacific Coast (Ref=Atlantic Coast) | 2.14 | 0.41 | 6.47 |  | 0.10 | -0.20 | 0.37 |
|  |  | Pacific Coast (Ref=Gulf Coast) | 1.29 | 0.15 | 4.84 |  | -0.03 | -0.37 | 0.31 |
|  | *V. cholerae* | | | | | | | | |
|  |  | Coastal (Ref=Non-Coastal) | 2.36 | 1.27 | 4.04 |  | 0.20 | 0.06 | 0.33 |
|  |  | Atlantic Coast (Ref=Non-Coastal) | 1.36 | 0.63 | 2.63 |  | 0.06 | -0.11 | 0.23 |
|  |  | Gulf Coast (Ref=Non-Coastal) | 26.23 | 7.06 | 76.43 |  | 0.55 | 0.40 | 0.67 |
|  |  | Pacific Coast (Ref=Non-Coastal) | 3.17 | 0.94 | 8.04 |  | 0.24 | -0.01 | 0.46 |
|  |  | Gulf Coast (Ref=Atlantic Coast) | 20.78 | 5.06 | 62.55 |  | 0.49 | 0.32 | 0.65 |
|  |  | Pacific Coast (Ref=Atlantic Coast) | 2.50 | 0.72 | 6.61 |  | 0.18 | -0.08 | 0.42 |
|  |  | Pacific Coast (Ref=Gulf Coast) | 0.17 | 0.03 | 0.53 |  | -0.31 | -0.56 | -0.09 |
|  | *V. fluvialis* | | | | | | | | |
|  |  | Coastal (Ref=Non-Coastal) | 8.49 | 4.75 | 14.59 |  | 0.38 | 0.27 | 0.49 |
|  |  | Atlantic Coast (Ref=Non-Coastal) | 7.26 | 3.50 | 13.77 |  | 0.34 | 0.19 | 0.49 |
|  |  | Gulf Coast (Ref=Non-Coastal) | 42.81 | 12.96 | 106.83 |  | 0.69 | 0.51 | 0.82 |
|  |  | Pacific Coast (Ref=Non-Coastal) | 3.74 | 1.12 | 9.00 |  | 0.18 | 0.01 | 0.40 |
|  |  | Gulf Coast (Ref=Atlantic Coast) | 6.35 | 1.83 | 16.75 |  | 0.36 | 0.14 | 0.55 |
|  |  | Pacific Coast (Ref=Atlantic Coast) | 0.56 | 0.16 | 1.40 |  | -0.16 | -0.37 | 0.08 |
|  |  | Pacific Coast (Ref=Gulf Coast) | 0.11 | 0.02 | 0.33 |  | -0.52 | -0.73 | -0.25 |
|  | *V. mimicus* | | | | | | | | |
|  |  | Coastal (Ref=Non-Coastal) | 7.70 | 3.99 | 14.16 |  | 0.17 | 0.10 | 0.23 |
|  |  | Atlantic Coast (Ref=Non-Coastal) | 3.27 | 1.46 | 6.67 |  | 0.06 | 0.02 | 0.13 |
|  |  | Gulf Coast (Ref=Non-Coastal) | 53.30 | 16.92 | 138.70 |  | 0.57 | 0.35 | 0.77 |
|  |  | Pacific Coast (Ref=Non-Coastal) | 2.89 | 0.80 | 8.37 |  | 0.05 | -0.01 | 0.17 |
|  |  | Gulf Coast (Ref=Atlantic Coast) | 17.71 | 5.24 | 45.41 |  | 0.50 | 0.29 | 0.71 |
|  |  | Pacific Coast (Ref=Atlantic Coast) | 0.95 | 0.25 | 2.61 |  | -0.01 | -0.10 | 0.10 |
|  |  | Pacific Coast (Ref=Gulf Coast) | 0.07 | 0.01 | 0.21 |  | -0.52 | -0.73 | -0.28 |
|  | *V. parahaemolyticus* | | | | | | | | |
|  |  | Coastal (Ref=Non-Coastal) | 18.70 | 7.04 | 41.21 |  | 0.47 | 0.32 | 0.62 |
|  |  | Atlantic Coast (Ref=Non-Coastal) | 12.09 | 4.11 | 28.60 |  | 0.43 | 0.27 | 0.59 |
|  |  | Gulf Coast (Ref=Non-Coastal) | 567.88 | 79.30 | 2125.33 |  | 0.54 | 0.38 | 0.70 |
|  |  | Pacific Coast (Ref=Non-Coastal) | 60.02 | 10.26 | 193.58 |  | 0.51 | 0.35 | 0.66 |
|  |  | Gulf Coast (Ref=Atlantic Coast) | 53.50 | 7.03 | 207.31 |  | 0.10 | 0.04 | 0.21 |
|  |  | Pacific Coast (Ref=Atlantic Coast) | 5.81 | 0.93 | 20.23 |  | 0.07 | -0.01 | 0.18 |
|  |  | Pacific Coast (Ref=Gulf Coast) | 0.20 | 0.02 | 0.84 |  | -0.03 | -0.09 | 0.00 |
|  | *V. vulnificus* | | | | | | | | |
|  |  | Coastal (Ref=Non-Coastal) | 9.06 | 4.00 | 18.75 |  | 0.45 | 0.30 | 0.59 |
|  |  | Atlantic Coast (Ref=Non-Coastal) | 7.02 | 2.53 | 16.16 |  | 0.38 | 0.18 | 0.57 |
|  |  | Gulf Coast (Ref=Non-Coastal) | 1276.23 | 113.70 | 6112.50 |  | 0.82 | 0.73 | 0.90 |
|  |  | Pacific Coast (Ref=Non-Coastal) | 5.01 | 0.99 | 16.12 |  | 0.27 | 0.00 | 0.58 |
|  |  | Gulf Coast (Ref=Atlantic Coast) | 210.55 | 17.98 | 931.79 |  | 0.45 | 0.26 | 0.64 |
|  |  | Pacific Coast (Ref=Atlantic Coast) | 0.82 | 0.14 | 2.70 |  | -0.11 | -0.42 | 0.24 |
|  |  | Pacific Coast (Ref=Gulf Coast) | 0.01 | 0.00 | 0.04 |  | -0.56 | -0.83 | -0.26 |
